# Prescribed daily-dose–based metrics of oral antibiotic use for hospitalized children in Japan

**DOI:** 10.1017/ash.2023.2

**Published:** 2023-02-02

**Authors:** Wataru Mimura, Daisuke Shinjo, Kensuke Shoji, Kiyohide Fushimi

**Affiliations:** 1 Department of Health Policy and Informatics, Tokyo Medical and Dental University Graduate School, Tokyo, Japan; 2 Division of Infectious Diseases, National Center for Child Health and Development, Tokyo, Japan

## Abstract

**Objective:**

Defined daily-dose (DDD)–based metrics are frequently used to measure antibiotic consumption. However, they are unsuitable for the pediatric population because they are defined using the maintenance dose for 70-kg adults. Moreover, children have large body weight variations. We assessed the prescribed daily dose (PDD) and PDD-based metrics of oral antibiotics for children to develop an alternative to DDD-based metrics in Japan.

**Design:**

We performed observational study using data from the Japanese administrative claims database between April 2018 and March 2019.

**Methods:**

Of 453,001 patients (aged 1 month–15 years), 564,326 admissions to 1,159 hospitals were included. We showed the median PDD (mg/day and mg/kg/day) and PDD-based metrics for 8 antibiotics for each age category (1 month to <1 year old and 1–6, 7–12, and 13–15 years old). We also assessed the relationship between PDD-based metrics and days of therapy (DOT)–based metrics using a scatter plot and correlation.

**Results:**

In total, 86,389 patients (19.1%) were prescribed oral antibiotics; amoxicillin, macrolides, and third-generation cephalosporins were the most common. The PDD (mg/day) for each antibiotic increased with age to 7–12 years old, when an adult dose was reached. The PDD (mg/kg/day) decreased with age to 13–15 years old, due to increasing body weight. The relationship between PDD per 1,000 patient days and DOT per 1,000 patient days differed depending on the antibiotic.

**Conclusions:**

PDD-based metrics stratified by age could characterize antibiotic consumption, even with body-weight variations. Therefore, PDD-based metrics, in addition to DOT-based metrics, are helpful benchmarks for antibiotic use in children.

A defined daily dose (DDD) has been commonly used in previous studies to quantify antibiotic consumption.^1–4^ DDDs are described by the World Health Organization as the maintenance dose per day for its main indication in 70 kg-adults.^5^ DDD-based metrics allowed us to compare the use of antibiotics by standardization, even in different countries, areas, or hospitals. However, applying DDDs to the child population is unsuitable due to variations in body weight. Instead, days of therapy (DOT), defined as the total number of days that patients were administered antibiotics regardless of the dose, has been used.^6–8^ Using the DOT metric has advantages and disadvantages; while it is unaffected by the changes and inconsistencies of DDDs, it cannot account for dose variations.^9,10^

DOT is the primary benchmark for monitoring the use of antibiotics and assessing antibiotic resistance in children. However, there are alternative metrics to quantify the dose of antibiotics, such as neonate DDDs, recommended daily doses per 100 kg days, and the prescribed daily dose (PDD).^2,11–16^ The PDD is based on data that reflect the prescribed dose. To deal with body weight variations, some studies have utilized PDDs as PDD (mg/kg/day) or have combined PDD (mg/day) with body weight stratification.^17–20^ PDDs are flexible and helpful metrics, even with weight variations similar to those in the child population. However, this metric must be developed in each country because of differences in indications or recommendations.

Several studies regarding the consumption of antibiotics have been conducted in the pediatric field since the antimicrobial resistance action plan was adopted in 2016.^21–27^ These studies are important for comparing differences between area or hospitalization and evaluating associations between consumption and antimicrobial resistance. Most studies have used DOT-based metrics to assess antibiotics; however, data regarding additional metrics that should be used within the child population are insufficient in Japan. Therefore, to develop a PDD methodology in Japan, we examined PDDs and assessed the relationship between PDD- and DOT-based metrics using a national administrative claims database.

## Methods

### Study design and setting

In this observational study, we used the Japanese Diagnosis Procedure Combination (DPC) data from April 2018 to March 2019. The DPC is a case-mix patient classification system that was adopted by ∼1,700 hospitals by 2018.^28–30^ The data consist of discharge abstracts; administrative claims data from acute-care hospitals and patient background information including age, sex, *International Classification of Diseases, Tenth Revision* diagnosis codes, procedures, and medications. The study included patients aged <16 years at admission. Neonates aged 28 days and younger were excluded because we focused on the assessment of oral antibiotics. We excluded admissions with body-weight outliers due to data-entry errors by using a mean ±3 standard deviation (SD) cutoff for every 1 year of age. To adjust for a large body-weight distribution, we categorized patient admissions into 4 age groups: 1 month to <1 year, 1–6 years, 7–12 years, and 13–15 years. We included all hospitalized patients throughout the year; thus, some patients were classified into 2 groups.

This study was approved by the Institutional Review Board of Tokyo Medical and Dental University. In addition, we used anonymized data for the studies; thus, the requirement for informed consent was waived.

### Variable definition

Age and body weight were recorded at admission. Ages were summarized as days in the 1 month to <1 year group and as years in the remaining 3 age groups. We used the medical diagnostic categories (MDCs) to indicate the inpatient diagnoses.^30^ The MDC system comprises 18 categories corresponding to a single organ system or etiology. Patients are assigned a 14-digit code related to the diagnosis of the most-used medical resources.

In Japan, the ratio of amoxicillin and clavulanic acid in the tablet form of amoxicillin-clavulanic acid (Augmentin) is 2:1, which is different from the international standard ratio of 7:1. Therefore, clinicians occasionally prescribe amoxicillin and amoxicillin-clavulanic acid simultaneously to adjust the ratio. We classified the medication as amoxicillin-clavulanic acid when amoxicillin and amoxicillin-clavulanic acid were administered on the same day. The PDD of amoxicillin-clavulanic acid was shown as an amoxicillin dose, and sulfamethoxazole and trimethoprim were shown as trimethoprim doses.

### Statistical analysis

We assessed patient age (days or years), sex, body weight, length of stay (LOS), and MDC in each age category. Continuous variables are shown as the median and interquartile range (IQR) or mean and SD, as appropriate. Categorical variables are presented as frequencies and percentages. We assessed the PDD metrics in 3 steps based on a previous study.^18^ First, we summarized the proportion of each antibiotic among patients who were prescribed oral antibiotics. We did not assess parenteral antibiotics because the data would reflect the dose for payment rather than the administered dose based on body weight. Second, we calculated median PDDs (mg/day and mg/kg/day) and interquartile ranges for frequently prescribed antibiotics, to deal with outliers. Third, we standardized the antibiotic use in each hospital using the median PDDs (mg/day and mg/kg/day) stratified by age group, as follows: (1) PDD per 1,000 patient days based on the median PDD (mg/day) and (2) PDD per 1,000 patient days based on the median PDD (mg/kg/day). In the latter metric, PDD per 1,000 patient days standardized using PDD (mg/day) was calculated as the median PDD (mg/kg/day) × body weight (kg); thus, both metrics of PDD per 1,000 patient days were unified to compare them with each other. In addition, we showed the differences between the 2 metrics, since PDD per 1,000 patient days by median PDD (mg/day) may not reflect the dosing variation associated with body weight, compared with PDD per 1,000 patient days by median PDD (mg/kg/day). Fourth, we compared hospital-level metrics, estimated as PDD per 1,000 patient days by median PDD (mg/kg/day) and DOT per 1,000 patient days, from hospitals that showed >30 days of therapy for each antibiotic. In addition, we confirmed the relationship between the PDD- and DOT-based methods using a scatter plot and Spearman correlation coefficients. Statistical significance was set at *P* < .05.

In this study, we evaluated several antibiotics that were highly ranked according to the first step. We excluded the data of doses >10 times the median PDD for each antibiotic when calculating the PDD per 1,000 patient days based on the median PDD (mg/kg/day) and DOT per 1,000 patient days. All analyses were performed using R version 4.0.5 software (The R Foundation for Statistical Computing, Vienna, Austria).

## Results

**Fig. 1.**
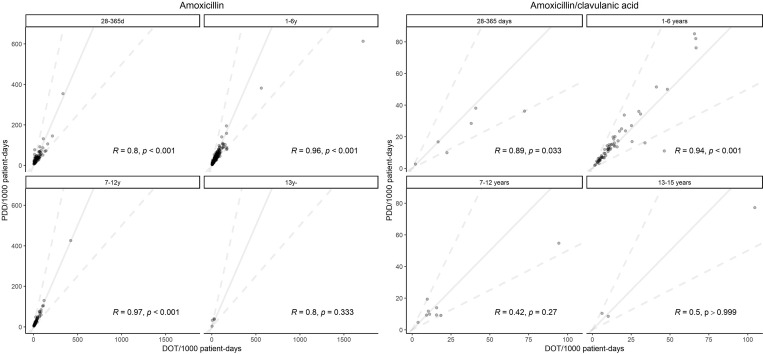
The relationship between DOT per 1,000 patient days and PDD per 1,000 patient days per hospital for amoxicillin and amoxicillin-clavulanic acid. The solid line represents a slope of 1; the upper dot line represents slope of 2, and the upper dot line represents slope of 0.5. PDD per 1,000 patient days based on the median PDD (mg/kg/day).

Among 465,407 patients and 579,417 admissions between April 2018 and March 2019, we included 453,001 patients and 564,326 admissions. In total, 12,406 patients (2.7%) and 1,5091admissions (2.6%) were excluded by the body weight outlier or mission data. Table 1 shows the characteristics of admitted patients in each age group, and the mean age and mean weight were 5 years (SD, 4) and 19 kg (SD, 14), respectively. The most common MDCs in the 1 month to <1 year group and the 1–6 years group were MDC04 (diseases and disorders of the respiratory system, mainly lower respiratory infections, influenza, and pneumonia; 40%) and MDC04 (diseases and disorders of the respiratory system, such as respiratory infections and asthma; 28%), respectively. In the 7–12 and 13–15 years old groups, the common diseases were MDC06 (diseases and disorders of the digestive system, hepatobiliary system, and pancreas, especially appendicitis, viral, and other specified intestinal infections, and inguinal hernia; 16%) and MDC16 (trauma, burns, and poisoning, including any type of fracture and injury; 22%), respectively. For the 86,389 patients (19.1%) that were prescribed oral antibiotics, the commonly prescribed oral antibiotics ranked as follows: amoxicillin (21.8%), clarithromycin (16.8%), cefditoren (12.7%), cefcapene (10.2%), cefaclor (9.4%), cefdinir (6.9%), sulfamethoxazole and trimethoprim (6.7%), amoxicillin-clavulanic acid (4.7%), and azithromycin (4.4%) (Supplementary Table 1). We assessed the PDD for the following 8 antibiotics: amoxicillin, amoxicillin-clavulanic acid, cefalexin, cefaclor, cefditoren, clarithromycin, tosufloxacin, and sulfamethoxazole and trimethoprim. The median PDDs (mg/day) were increased in the 1 month to <1 year group and the 7–12 years group, and it did not increase in the 7–12 years group or the 13–15 years group for all antibiotics (Table 2). Regarding PDDs (mg/kg/day), there were no significant differences between the 1 month to <1 year group and the 1–6 years group; however, they were gradually decreased in the 7–12 years group. After the use of antibiotic was standardized using the median PDD (mg/day) and median PDD (mg/kg/day), no differences were observed between PDD per 1,000 patient days based on the median PDD (mg/day) and PDD per 1,000 patient days based on the median PDD (mg/kg/day) (Table 3). The correlation coefficients were high for most antibiotics; however, the relationship between PDD per 1,000 patient days based on the median PDD (mg/kg/day) and DOT per 1,000 patient days differed between antibiotics (Fig. 1 and Supplementary Material).

**Table 1.** Admission Characteristics for Pediatric Patients in Each Age Category

Characteristic	1 Month to <1 Year (N = 92,976)	1–6 Years (N = 306,770)	7–12 Years (N = 111,606)	13–15 Years (N = 52,974)
Age, mean (SD)^a^	188 (105)	3 (2)	9 (2)	14 (1)
Sex, male	54,092 (58)	173,908 (57)	64,186 (58)	30,352 (57)
Body weight (kg), mean (SD)	7 (2)	13 (4)	30 (10)	49 (12)
LOS (days), median (IQR)	5 (3–7)	4 (3–6)	4 (2–7)	5 (3–9)
**MDC classification, n (%)**				
Nervous system	3,479 (3.7)	14,285 (4.7)	8,871 (7.9)	4,435 (8.4)
Eye	648 (0.7)	4,290 (1.4)	3,212 (2.9)	1,248 (2.4)
Ear, nose, and throat	8,088 (8.7)	31,638 (10)	10,134 (9.1)	2,861 (5.4)
Respiratory system	36,910 (40)	87,175 (28)	12,299 (11)	3,540 (6.7)
Circulatory system	513 (0.6)	1,085 (0.4)	1,243 (1.1)	1,443 (2.7)
Digestive system, hepatobiliary system, and pancreas	8,127 (8.7)	34,955 (11)	17,447 (16)	8,277 (16)
Musculoskeletal system and connective tissues	1,010 (1.1)	6,336 (2.1)	5,464 (4.9)	4,476 (8.4)
Skin and subcutaneous tissue	5,017 (5.4)	37,419 (12)	10,628 (9.5)	2,595 (4.9)
Breast	6 (<0.1)	14 (<0.1)	27 (<0.1)	55 (0.1)
Endocrine, nutritional, and metabolic system	1,891 (2.0)	13,103 (4.3)	8,025 (7.2)	2,995 (5.7)
Kidney, urinary tract, and male reproductive system	6,465 (7.0)	7,753 (2.5)	5,099 (4.6)	2,398 (4.5)
Female reproductive system, pregnancy, childbirth, and puerperium	46 (<0.1)	147 (<0.1)	378 (0.3)	676 (1.3)
Blood, blood-forming organ and myeloproliferative diseases and disorders	848 (0.9)	7,613 (2.5)	4,372 (3.9)	1,841 (3.5)
Neonatal diseases and disorders	10,943 (12)	21,155 (6.9)	6,256 (5.6)	2,118 (4.0)
Pediatric diseases and disorders	3,904 (4.2)	23,110 (7.5)	1,624 (1.5)	249 (0.5)
Trauma, burns, and poisonings	1,804 (1.9)	10,753 (3.5)	14,130 (13)	11,864 (22)
Mental diseases and disorders	142 (0.2)	956 (0.3)	677 (0.6)	1,108 (2.1)
Other diseases and disorders	3,123 (3.4)	4,970 (1.6)	1,707 (1.5)	775 (1.5)

Note. IQR, interquartile range; LOS, length of stay; MDC, major diagnosis category; SD, standard deviation.

a The age presented as days in 1 month to <1 year, others presented as years.

**Table 2.** Prescribed Daily Dose (PDD, mg/day and mg/kg/day) in 4 Age Groups

Antibiotic	1 Month to <1 Years Median (IQR)	1–6 Years Median (IQR)	7–12 Years Median (IQR)	13–15 Years Median (IQR)
**Amoxicillin**				
PDD (mg/day)	300 (200–400)	460 (360–600)	750 (650–840)	750 (750–900)
PDD (mg/kg/day)	39.0 (30.0–49.0)	37.7 (30.0–43.1)	27.8 (20.7–33.3)	16.0 (12.6–23.0)
**Amoxicillin-clavulanic acid^a^**				
PDD (mg/day)	600 (600–600)	1,188 (600–1,200)	1,500 (750–2,400)	1,500 (750–1,500)
PDD (mg/kg/day)	84.1 (70.6–100.0)	83.3 (64.8–96.8)	75.6 (25.1–89.9)	24.2 (14.8, 39.0)
**Cefalexin**				
PDD (mg/day)	360 (260–450)	600 (450–900)	1,000 (750–1,500)	1,000 (750–1,500)
PDD (mg/kg/day)	50.0 (42.7–67.6)	40.5 (31.0–51.1)	30.4 (22.5–48.5)	19.1 (14.7–30.2)
**Cefaclor**				
PDD (mg/day)	180 (80–250)	300 (170–450)	750 (250–750)	750 (750–750)
PDD (mg/kg/day)	30.0 (11.8–37.5)	28.2 (11.7–31.6)	19.4 (9.5–27.3)	15.0 (12.7–17.7)
**Cefditoren**				
PDD (mg/day)	70 (60–90)	135 (100–170)	252 (200–300)	300 (200–300)
PDD (mg/kg/day)	9.4 (8.8–10.5)	9.2 (8.8–10.0)	8.7 (7.5–9.4)	5.7 (3.7–6.7)
**Clarithromycin**				
PDD (mg/day)	60 (30–97)	130 (70–190)	200 (100–400)	200 (100–400)
PDD (mg/kg/day)	10.8 (5.5–14.3)	10.9 (5.6–14.4)	8.3 (4.6–12.0)	5.2 (3.6–8.3)
**Tosufloxacin**				
PDD (mg/day)	98 (84–110)	160 (120–200)	300 (240–340)	300 (300–360)
PDD (mg/kg/day)	12.0 (11.5–12.9)	12.0 (11.5–12.3)	11.6 (10.2–12.1)	8.0 (6.2–10.1)
**Sulfamethoxazole and trimethoprim^b^**				
PDD (mg/day)	32 (22–48)	72 (40–80)	144 (80–160)	160 (80–160)
PDD (mg/kg/day)	5.4 (3.6–7.7)	4.9 (3.6–6.9)	4.3 (3.0–6.0)	3.1 (1.9–4.8)

Note. IQR, interquartile range.

a Amoxicillin-clavulanic acid presented as the amoxicillin dose.

b Sulfamethoxazole and trimethoprim presented as the trimethoprim dose.

**Table 3.** Comparison of Prescribed Daily Dose (PDD) per 1,000 Patient Days Based on Median PDD (mg/day and mg/kg/day)

Antibiotic	No. of Groups^a^	PDD/1,000 Patient Days, by Median PDD Mean mg/day (SD)	PDD/1,000 Patient Days by Median PDD Mean mg/kg/day (SD)	Difference Mean (SD)
Amoxicillin	470	41.2 (71.4)	42.9 (90.1)	−1.7 (22)
Amoxicillin/clavulanic acid	78	20.9 (30.2)	18.4 (20.7)	2.5 (16)
Cefalexin	49	32 (42.4)	28.9 (41.1)	3.2 (7.3)
Cefaclor	213	29.4 (42.2)	31.8 (51.2)	−2.4 (13.7)
Cefditoren	273	34.2 (36.6)	34.1 (39.8)	0.1 (6.5)
Clarithromycin	688	59.5 (68.7)	65.1 (78.8)	−5.5 (16.8)
Tosufloxacin	151	38.6 (52.8)	39 (51)	−0.4 (8)
Sulfamethoxazole and trimethoprim	513	106 (101.5)	94 (91.2)	12 (28.2)

a The groups included >30 days of therapy per age group.

## Discussion

In this study, we developed a PDD-based methodology to assess the use of antibiotics in children. We showed median PDDs (mg/day and mg/kg/day) and the relationship between DOT per 1,000 patient days and PDD per 1,000 patient days for pediatric patients using the nationwide inpatient database in Japan. In addition, we characterized the relationship between PDD (mg/day) and DOT metrics and used scatter plots to describe the variation between the hospitals. We found that PDD (mg/day) metrics-stratified age groups could be an alternative to DDD-based metrics in the pediatric population.

Our results for oral antibiotic prescriptions for pediatric inpatients showed a similar tendency to that of pediatric outpatients in Japan.^22–24^ These studies also reported that penicillin with an extended-spectrum (anatomical therapeutic chemical code: J01CA), third-generation cephalosporins (J01DD), and macrolides (J01FA) were commonly prescribed for several years. In addition to the prevalence of antibiotics, we calculated PDDs for each antibiotic. The median PDD (mg/day) increased gradually with increasing age, reaching a plateau at 7–12 years for each antibiotic. In contrast, PDDs (mg/kg/day) decreased for each antibiotic because the patients were more likely to reach an adult body weight; thus, the median PDDs (mg/day) gradually decreased with increasing age.

For amoxicillin and amoxicillin-clavulanic acid, the PDDs showed a significant difference in the amoxicillin dose (Table 2). The PDDs of amoxicillin-clavulanic acid were more than double that of the PDDs of amoxicillin in all groups. These doses coincide with the Japanese guidelines for respiratory disease and labeled doses in Japan, and these variations may reflect the indications for each antibiotic.^31^ In our study, we indicated the PDDs as an amoxicillin dose instead of as a unit dose because the ratios of amoxicillin-clavulanic acid differed depending on the product used^32^; some are at a ratio of 2:1 and others are 14:1. In addition, clinicians occasionally prescribe amoxicillin-clavulanic acid products with amoxicillin products to adjust the ratio. Porta et al^18^ reported that the PDDs (mg/kg/day) of amoxicillin plus clavulanic acid varied widely between centers, even though it was commonly prescribed in each hospital. These variations are explained mainly by differences in the disease or indications, which characterize the use of antibiotics.

The relationship between PDD per 1,000 patient days and DOT per 1,000 patient days varied with the antibiotics. For example, for tosufloxacin, the 2 metrics were almost identical (Supplementary Fig. 1). The study also reported a similar tendency for the difference between DDD per 1,000 patient days and DOT per 1,000 patient days for levofloxacin.^9^ In contrast, clarithromycin and sulfamethoxazole and trimethoprim were widely distributed. The results were associated with the wide range of doses. Sulfamethoxazole and trimethoprim are used to prevent urinary tract infections in children with vesicoureteral reflux or *Pneumocystis* pneumonia in children with immune suppression.^33,34^ Macrolides also have low-dose treatment options for chronic airway diseases.^35^ Thus, PDD-based metrics are more likely to be beneficial than DOT-based metrics for assessing the consumption of antibiotics when the range of doses is wide due to indications. The results imply that the PDD and DOT methodologies have different characteristics as metrics, and combining the 2 metrics was beneficial to audit child antibiotic use.

We used the median PDDs (mg/day) with stratification by age instead of the median PDDs (mg/kg/day) with body weight to adjust the dosing variation due to body weight. The stratification by age were valuable to control variations because the differences between the 2 metrics of PDD per 1,000 patient days may not be significant. Using the median PDDs (mg/day) in the stratified cohort is acceptable, given that DDDs are defined by the dose in 70-kg adults. Furthermore, there is a benefit to using the PDDs (mg/day) stratified by age because there are limitations to obtaining information on patient body weight in Japan. The Japanese claims database, which is mainly used to evaluate oral antibiotics in an outpatient setting, does not generally contain body-weight data, but it includes age. Thus, using PDD (mg/day) after stratifying by age would be one of the most practical approaches to assess the consumption of antibiotics rather than PDD (mg/kg/day).

The *Manual of Antimicrobial Stewardship* and changes in the reimbursement system regarding the regulation of antimicrobial stewardship fees after the national action plan for antimicrobial resistance were adopted in Japan.^21,36^ Studies have reported that after the new antimicrobial stewardship fee was implemented, the frequency of antibiotic prescriptions decreased.^37–39^ These changes were made to promote appropriate antimicrobial therapy and affect decision making regarding the necessity of treatment, antibiotic selection, treatment period, and dose. Monitoring PDDs and the use of PDD benchmarking allows us to assess the effects in detail.

This study has notable strengths. It is the largest reported study on this subject based on a national administrative database. In addition, we have developed metrics for the use of antibiotics in Japanese pediatric settings, and we have assessed hospital variations for PDD- and DOT-based metrics. Our results indicate that the use of PDD- and DOT-based metrics, instead of DDD-based metrics, is helpful in pediatric settings and informs future interventions to improve the use of antibiotics in children. The metrics can also be used for benchmarking hospitals and further longitudinal analyses based on the database. Moreover, DDD-based metrics are not suitable for patients with renal dysfunction and children.^9^ Therefore, the potential application of PDD-based metrics should be examined by utilizing more detailed data, such as electronic health records, in further studies including patients and populations that reported large deviations between PDD and DDD.^19^

This study had several limitations. First, these metrics cannot be applied to children in other countries because the PDDs indicate the dose prescribed for pediatric patients in Japan. In addition, our findings may not be generalizable to other populations, although we used the nationwide database in Japan. We could not assess the validity in outpatients due to the use of inpatient data. Second, we excluded admissions with an outlier or missing values of body weight due to data-entry errors. However, median PDD (mg/day) and PDD (mg/kg/day) values could be reasonable under the approved dosage and indications for infectious disease in Japan. Third, we could not show the PDD for parenteral antibiotics because the data more likely reflected the dose for payments and not the dose received by patients. Thus, these data were not suitable for PDD, and we alternative methods or data are needed to assess the dose of parenteral antibiotics in addition to DOT metrics in pediatric patients.

In conclusion, we assessed PDD (mg/day and mg/kg/day) and oral antibiotic metrics among children admitted to hospitals in Japan. The PDDs (mg/day) and PDDs (mg/kg/day) changed gradually, increasing with age. In addition, the relationship between the PDD per 1,000 patient days and DOT per 1,000 patient days was antibiotic dependent. Therefore, PDD could be useful for auditing intra- and interhospital changes and differences in antibiotic usage in Japan.

## Supplementary material

For supplementary material accompanying this paper visit http://doi.org/10.1017/ash.2023.2. click here to view supplementary material
