# Supplementary material for: Prescribed daily-dose–based metrics of oral antibiotic use for hospitalized children in Japan
Source: Antimicrob Steward Healthc Epidemiol. 2023 Feb 2;3(1):e24. doi: 10.1017/ash.2023.2 (PMC9932642; doi:10.1017/ash.2023.2)
Supplement: Supplementary file 1 [file S2732494X23000025sup.zip › S2732494X23000025sup001.docx]

**Supplementary Table 1. The proportion of each antibiotic in patients who prescribes any oral antibiotics.**

| ATC code | Antibiotic name | N = 86389 | DDD (unit) |
| --- | --- | --- | --- |
| J01AA02 | Doxycycline | 51 (0.1) | 0.1 (g) |
| J01AA07 | Tetracycline | 43 (0) | 1 (g) |
| J01AA08 | Minocycline | 1230 (1.4) | 0.2 (g) |
| J01CA01 | Ampicillin | 180 (0.2) | 2 (g) |
| J01CA04 | Amoxicillin | 19264 (21.8) | 1.5 (g) |
| J01CE08 | Benzylpenicillin | 10 (0) | 3.6 (g) |
| J01CR02 | Amoxicillin/clavulanate | 4104 (4.7) | 1.5 (g) |
| J01CR04 | Sultamicillin | 610 (0.7) | 1.5 (g) |
| J01CR50 | Ampicillin/cloxacillin | 1 (0) | - |
| J01DB01 | Cefalexin | 2314 (2.6) | 2 (g) |
| J01DB11 | Cefroxadine | 117 (0.1) | 2.1 (g) |
| J01DC02 | Cefuroxime | 10 (0) | 0.5 (g) |
| J01DC04 | Cefaclor | 8328 (9.4) | 1 (g) |
| J01DD08 | Cefixime | 76 (0.1) | 0.4 (g) |
| J01DD13 | Cefpodoxime | 624 (0.7) | 0.4 (g) |
| J01DD15 | Cefdinir | 6066 (6.9) | 0.6 (g) |
| J01DD16 | Cefditoren | 11238 (12.7) | 0.4 (g) |
| J01DD17 | Cefcapene | 9010 (10.2) | 0.45 (g) |
| J01DD18 | Cefteram | 95 (0.1) | 0.4 (g) |
| J01DH06 | Tebipenem | 173 (0.2) | 0.56 (g) |
| J01DI03 | Faropenem | 284 (0.3) | 0.75 (g) |
| J01EE01 | Sulfamethoxazole and trimethoprim | 5912 (6.7) | 4 (UD) |
| J01FA01 | Erythromycin | 1552 (1.8) | 1 (g) |
| J01FA06 | Roxithromycin | 38 (0) | 0.3 (g) |
| J01FA07 | Josamycin | 1 (0) | 2 (g) |
| J01FA09 | Clarithromycin | 14822 (16.8) | 0.5 (g) |
| J01FA10 | Azithromycin | 3855 (4.4) | 0.3 (g) |
| J01FF01 | Clindamycin | 160 (0.2) | 1.2 (g) |
| J01MA01 | Ofloxacin | 1 (0) | 0.4 (g) |
| J01MA02 | Ciprofloxacin | 76 (0.1) | 1 (g) |
| J01MA06 | Norfloxacin | 74 (0.1) | 0.8 (g) |
| J01MA12 | Levofloxacin | 605 (0.7) | 0.5 (g) |
| J01MA14 | Moxifloxacin | 3 (0) | 0.4 (g) |
| J01MA17 | Prulifloxacin | 5 (0) | 0.6 (g) |
| J01MA19 | Garenoxacin | 49 (0.1) | 0.4 (g) |
| J01MA21 | Sitafloxacin | 9 (0) | 0.1 (g) |
| J01MA22 | Tosufloxacin | 3740 (4.2) | 0.45 (g) |
| J01XX01 | Fosfomycin | 2180 (2.5) | 3 (g) |
| J01XX08 | Linezolid | 75 (0.1) | 1.2 (g) |

Supplementary Table 2. Admission characteristics for pediatric patients prescribed oral antibiotics in each age categories

| Characteristic | 1 month to <1 years | 1–6 years | 7–12 years | 13–15 years |
| --- | --- | --- | --- | --- |
|  | N = 17,870 | N = 69,767 | N = 25,792 | N = 11,546 |
| Age (days or years), mean (SD)^1^ | 206 (100) | 3 (2) | 9 (2) | 14 (1) |
| Sex (male) | 10,981 (61%) | 39,023 (56%) | 14,405 (56%) | 6,691 (58%) |
| Body weight (kg), mean (SD) | 7 (2) | 13 (4) | 30 (10) | 48 (13) |
| LOS (days), median (IQR) | 6 (5, 9) | 6 (4, 8) | 6 (3, 9) | 6 (4, 12) |
| MDC classification |  |  |  |  |
| Nervous System | 369 (2.1%) | 2,470 (3.5%) | 1,353 (5.2%) | 571 (4.9%) |
| Eye | 260 (1.5%) | 1,912 (2.7%) | 1,461 (5.7%) | 569 (4.9%) |
| Ear, Nose, and Throat | 1,111 (6.2%) | 9,843 (14%) | 3,812 (15%) | 918 (8.0%) |
| Respiratory System | 8,095 (45%) | 28,217 (40%) | 4,713 (18%) | 1,274 (11%) |
| Circulatory System | 59 (0.3%) | 253 (0.4%) | 170 (0.7%) | 181 (1.6%) |
| Digestive System, Hepatobiliary System, and Pancreas | 641 (3.6%) | 4,045 (5.8%) | 2,653 (10%) | 1,556 (13%) |
| Musculoskeletal System and Connective Tissues | 245 (1.4%) | 1,820 (2.6%) | 1,645 (6.4%) | 1,242 (11%) |
| Skin and Subcutaneous Tissue | 394 (2.2%) | 2,307 (3.3%) | 912 (3.5%) | 387 (3.4%) |
| Breast | 2 (<0.1%) | 3 (<0.1%) | 5 (<0.1%) | 8 (<0.1%) |
| Endocrine, Nutritional and Metabolic System | 216 (1.2%) | 1,768 (2.5%) | 794 (3.1%) | 290 (2.5%) |
| Kidney, Urinary Tract and Male Reproductive System | 3,294 (18%) | 3,064 (4.4%) | 1,897 (7.4%) | 866 (7.5%) |
| Female Reproductive System, Pregnancy, Childbirth, and Puerperium | 7 (<0.1%) | 22 (<0.1%) | 67 (0.3%) | 129 (1.1%) |
| Blood, Blood Forming Organ and Myeloproliferative Diseases and Disorders | 215 (1.2%) | 3,548 (5.1%) | 2,167 (8.4%) | 962 (8.3%) |
| Neonatal Diseases and Disorders | 2,183 (12%) | 5,768 (8.3%) | 1,750 (6.8%) | 538 (4.7%) |
| Pediatric Diseases and Disorders | 290 (1.6%) | 2,228 (3.2%) | 241 (0.9%) | 65 (0.6%) |
| Trauma, Burns, and Poisonings | 84 (0.5%) | 1,134 (1.6%) | 1,617 (6.3%) | 1,698 (15%) |
| Mental Diseases and Disorders | 2 (<0.1%) | 37 (<0.1%) | 41 (0.2%) | 57 (0.5%) |
| Other Diseases and Disorders | 403 (2.3%) | 1,325 (1.9%) | 494 (1.9%) | 233 (2.0%) |

IQR, interquartile range; LOS, length of stay; MDC, major diagnosis category.

1The age showed as age-days in 1 month–<1 years, others showed as age-years.

**Supplementary Figure legend**

**Supplementary Figure 1. The relationship between DOT/1000 patient-days and PDD/1000 patient-days per hospital for cefalexin, cefaclor, cefditoren, clarithromycin, tosufloxacin, and sulfamethoxazole and trimethoprim.**

The solid line showed the slope is 1; the upper dot line showed the slope is 2, and the upper dot line showed the slope is 0.5.
